# Supplementary material for: Consumer Perception and Liking of Parmigiano Reggiano Protected Designation of Origin (PDO) Cheese Produced with Milk from Cows Fed Fresh Forage vs. Dry Hay
Source: Foods. 2024 Jan 18;13(2):309. doi: 10.3390/foods13020309 (PMC10815482; doi:10.3390/foods13020309)
Supplement: Supplementary file 1 [file foods-13-00309-s001.zip › foods-2809868-supplementary.pdf]

Supplementary material

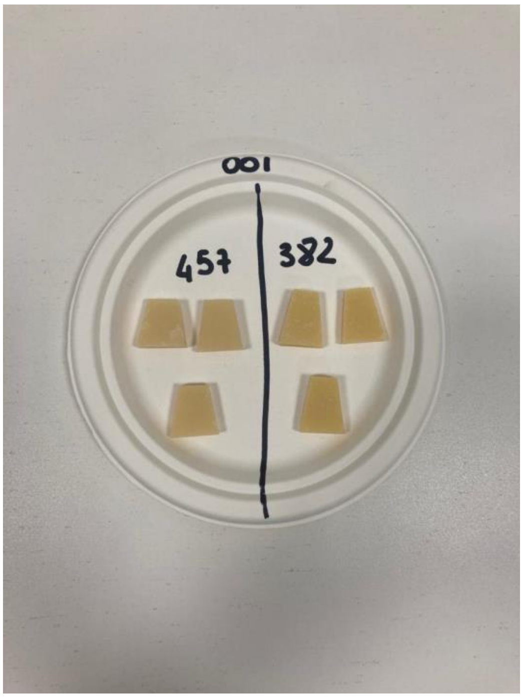


**Figure S1**. Example of sample presentation of Parmigiano Reggiano to consumers.
